# Single dendrite-targeting interneurons generate branch-specific inhibition

**DOI:** 10.3389/fncir.2014.00139

**Published:** 2014-11-25

**Authors:** Caleb C. A. Stokes, Corinne M. Teeter, Jeffry S. Isaacson

**Affiliations:** Department of Neuroscience, Center for Neural Circuits and Behavior, School of Medicine, University of CaliforniaSan Diego, La Jolla, CA, USA

**Keywords:** piriform cortex, olfactory cortex, backpropagating action potential, pyramidal cells, IPSP/C (inhibitory postsynaptic potential/current)

## Abstract

Microcircuits composed of dendrite-targeting inhibitory interneurons and pyramidal cells (PCs) are fundamental elements of cortical networks, however, the impact of individual interneurons on pyramidal dendrites is unclear. Here, we combine paired recordings and calcium imaging to determine the spatial domain over which single dendrite-targeting interneurons influence PCs in olfactory cortex. We show that a major action of individual interneurons is to inhibit dendrites in a branch-specific fashion.

## Introduction

GABAergic interneurons underlying cortical inhibition can be subdivided into classes based on the location of their synaptic targets: for example, those that form inhibitory synapses primarily on the perisomatic membrane of pyramidal cells (PCs) and those that preferentially contact dendrites (Markram et al., 2004). Interneurons targeting the cell body region (near the trigger zone for spike initiation) are poised to strongly regulate action potential (AP) output. Indeed, the strong somatic hyperpolarization generated by a single presynaptic basket cell is sufficient to regulate repetitive AP firing and phase PC activity (Cobb et al., 1995; Miles et al., 1996). Dendritic inhibition can dampen the passive propagation of excitatory synaptic input to the soma (Jack et al., 1975; Gidon and Segev, 2012) as well as modulate active forms of integration based on electrogenic properties of dendrites (dendritic Na^+^ or Ca^2+^ “spikes”) (Tsubokawa and Ross, 1996; Larkum et al., 1999; Lovett-Barron et al., 2012; Müller et al., 2012). Although it has been reported that activation of single interneurons can block generation of dendritic Ca^2+^ spikes in neocortical layer 5 PCs (Larkum et al., 1999), the functional role of individual dendrite-targeting interneurons is not well understood.

An important question is whether interneurons elicit dendritic inhibition globally or in a more local, branch-specific manner. Experiments using exogenously applied GABA or photoactivation of interneuron synapses suggest that dendritic inhibition can be highly focal, and even compartmentalized to the level of single dendritic spines (Kanemoto et al., 2011; Chiu et al., 2013). In contrast, theoretical work suggests that activation of multiple GABAergic synapses distributed across the PC dendritic tree will cause more global inhibition, with the effects of inhibition being maximal at dendritic locations that may be far from the actual sites of inhibitory synaptic connections (Gidon and Segev, 2012). Defining the spatial domain of inhibition is critical for understanding the computations performed by PC dendrites; however, the actions of single dendrite-targeting interneurons on dendritic excitability are not well understood.

Here, we take advantage of interneurons in layer 1a (L1 INTs) of piriform cortex, which only contact the distal apical dendrites of layer 2/3 PCs (Stokes and Isaacson, 2010; Suzuki and Bekkers, 2012). We use 2-photon calcium imaging and paired whole-cell recordings to investigate the spatial domain of inhibition produced by a single dendrite-targeting interneuron onto the apical dendrites of PCs.

## Materials and methods

### Slice preparation

All animal procedures were conducted in accordance with the US National Institutes of Health guidelines and with the approval of the Committee on Animal Care at the University of California, San Diego. Mice (C57bl/6, P15-P22) used were of both sexes and group-housed in the vivarium under were reversed light-dark (12 h and 12 h) conditions. The mice used had no previous history of drug administration, surgery or behavioral testing and were anesthetized with ketamine and xylazine (100 mg kg^−1^ and 10 mg kg^−1^, respectively). Olfactory cortex was cut into 400 μm parasagittal slices in ice-cold sucrose solution (in mM: NaCl, 83; KCl, 2.5; MgSO_4_, 3.3; NaH_2_PO_4_, 1; NaHCO_3_, 26.2; D-glucose, 22; sucrose, 72; and CaCl_2_, 0.5, bubbled with 95% O_2_ and 5% CO_2_). Slices were incubated in sucrose solution at 34°C for 30 min and then at room temperature (21°C) until used for recordings.

### Electrophysiology, simulations and imaging

Whole-cell recordings were performed at 28°–30°C in artificial cerebrospinal fluid containing (in mM): 119 NaCl, 2.5 KCl, 2.5 CaCl_2_, 1.3 MgSO_4_, 1 NaH_2_PO_4_, 26.2 NaHCO_3_ and 22 glucose, equilibrated with 95% O_2_ and 5% CO_2_. Cells were targeted for recordings based on their soma shape and location in layer 1 (interneurons) or layer 3 (pyramidal cells) and identity was confirmed based on intrinsic electrical properties and/or *post hoc* reconstructions. Paired cell recordings were made using a Multiclamp 700 B amplifier (Molecular Devices) and analyzed with Axograph X and Igor Pro software. Pipettes (3–5 MΩ) contained a potassium-based internal solution (in mM: 150 potassium gluconate, 1.5 MgCl_2_, 5 HEPES buffer, 10 phosphocreatine, and 2.0 Mg-ATP, adjusted to pH 7.4 with KOH, *E*_Cl_ ~−80 mV). Conductance was calculated by solving for G in voltage-clamp recordings of inhibitory current using *G* = IPSC/(*Vm*−*E*_Cl_) where *E*_Cl_ = −80 mV, as determined by voltage-clamp experiments using empirical measurement of the reversal potential. Series resistance was <20 MΩ. For anatomical reconstructions, 0.2% biocytin was added to the internal solution and cells revealed by a DAB reaction with nickel intensification. Cells were manually reconstructed and both neurite length and diameter measured using Neurolucida (MBF) with further analysis using Neurolucida Explorer (MBF). Putative synaptic contacts were identified based on the presence of both the close approximation of identified axonal and dendritic processes as well as presynaptic swelling of the axon consistent with a bouton.

To ensure that recordings were made from cells with intact structure we only targeted cells with somata located >100 μm below the slice surface. Additionally, we confirmed that the apical dendrites of L3 PCs were intact and overlapped with the field of L1 INT axons by performing 3D visualizations following reconstruction. Structural information from L3 PCs was imported from Neurolucida tracings into the NEURON simulation environment (Yale University). Values for axial resistance (173 Ω·cm), input resistance (23 kΩ·cm^2^), and capacitance (1.3 μF·cm^2^) were derived from previous measures of olfactory cortex PCs (Bathellier et al., 2009). Simulations were performed using a dynamic clamp configuration (Bagnall et al., 2011) and individual GABA_A_ synapses were modeled as a 0.1 nS conductance. This value produced somatic hyperpolarization equivalent to the recorded unitary synaptic potentials (~0.2 mV). Simulations using larger synaptic conductances (0.3–0.5 nS) revealed similar branch selective effects (not shown). Values for resting potential and Cl^−^ reversal potential were set at −70 and −80 mV, respectively.

For imaging Ca^2+^ transients associated with back-propagating APs in L3 PCs, the calcium indicator Oregon Green 488 BAPTA-1 (100 μM, Life Technologies) was included in the patch electrode. For some experiments examining the relationship between APs and dendritic calcium transients, imaging was performed with a cooled CCD camera imaging system (T.I.L.L. Photonics). All other imaging experiments were performed on a custom-built two-photon microscope (Olympus BX51WI) controlled by FluoView software (Olympus) using a laser (Spectra Physics) wavelength of 830 nm. In paired recordings, the L1 INT electrode contained the red fluorescent dye Alexa-594 (50 μM, Life Technologies). Line scans transecting 1–3 branches of the apical dendrite were performed at 200 Hz and trials with and without co-activation of L1 INTs were always interleaved. The onset of the train of L1 INT APs preceded the onset of L3 PC APs by 2 ms. DeltaF/F was determined from averages of 3–5 trials and analyzed using Igor Pro (Wavemetrics). Traces were filtered (binomial smoothing) and values of deltaF were determined from the peak amplitude of a first-order exponential fit to the falling phase of Ca^2+^ transients. Peak deltaF/F was highly reliable across trials both in the presence and absence of inhibition (standard deviation = 1–3%, not shown). Thus, significant inhibition of Ca^2+^ transients (*p* ≤ 0.05, student’s *t*-test) could be detected from 3–5 trials even when modulation was as small as 10–15%. Reference images of the entire L3 PC dendritic arbor derived from z-stacks were collected at the end of each experiment. Data were collected and analyzed without randomization or blinding.

## Results

**Figure 1:**
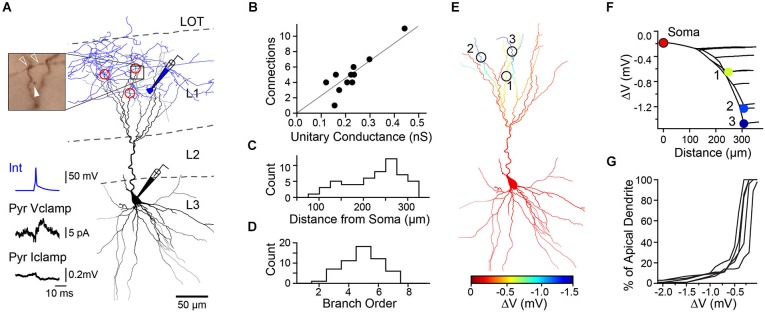
**A combination of dendritic synaptic location and L3 PC cable properties can produce branch specific inhibition by single L1 INTs. (A)** Anatomical reconstruction of a synaptically connected cell pair (black: PC soma and dendrites, blue: L1 INT soma and axon) and location of putative synaptic contacts (red circles). Upper left, light micrograph showing site of one synaptic contact (open arrowheads: INT axon boutons, closed arrowhead: PC dendritic spine). Bottom left, traces show PC (black) average unitary inhibitory postsynaptic current (Vclamp, *V*_h_ = −50 mV) and potential (Iclamp, resting potential) in response to an INT spike (blue). **(B)** Summary of results from reconstructed pairs (*n* = 11 cells, 11 slices, 11 mice) shows relationship between number of putative connections and measured unitary conductance. Line, linear fit, *r*^2^ = 0.79. **(C)** Location of synaptic contacts relative to PC soma. **(D)** Branch order of PC dendrites receiving synaptic contacts. **(E)** Simulated spatial profile of hyperpolarization from resting membrane potential (ΔV) for the PC recorded in **(A)**. **(F)** Plot of hyperpolarization vs. distance from soma (0 μm) for all regions of apical dendrite with numbered synaptic contacts corresponding to sites in **(E)**. Red circle at distance = 0 represents the voltage at the soma. **(G)** Cumulative distribution of predicted dendritic hyperpolarization for reconstructed L1 INT-L3 PC pairs (*n* = 5 cells, 5 slices, 5 mice).

We first determined the properties of unitary connections between L1 INTs and L3 PCs by recording from synaptically connected cell pairs in piriform cortex slices. *Post hoc* reconstructions of biocytin-filled cells were used to identify sites of putative synaptic contacts (Markram et al., 1997). Individual L1 INTs elicited only a weak hyperpolarization at the somatic recording location (mean = −0.13 ± 0.05 mV, average conductance = 0.22 ± 0.03 nS, *N* = 11, Figures 1A,B). Reconstructions of the same cells (Figures 1A–C) revealed that L1 INTs made sparse connections (average = 5, range 1–11) exclusively onto the distal apical dendrites of L3 PCs (>100 μm from soma, mean distance 226 ± 8 μm). Interneuron contacts were never observed on primary apical dendrites, rather the putative synaptic contacts were found on higher-order branches (mean branch order = 4.9 ± 0.2, Figure 1D).

**Figure 2:**
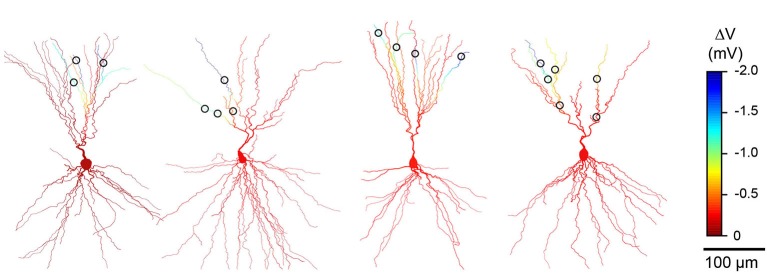
**Simulations consistently predict branch specific inhibition in different L3 pyramidal cells (PCs)**. Results from anatomical reconstructions of four additional cell pairs (as in Figure 1) showing dendritic morphology of L3 PCs and L1 interneuron synaptic contacts (circles). Color scale indicates simulated change in membrane potential during co-activation of all inhibitory synaptic contacts.

We next used computational modeling of reconstructed L3 PCs to examine how the passive cable properties of dendrites affect the distribution of hyperpolarization when sparsely distributed GABA_A_-receptor mediated inhibitory synapses are co-activated. Consistent with previous studies (Golding et al., 2005; Bathellier et al., 2009; Gidon and Segev, 2012), distal synapses caused local changes in dendritic membrane potential that were many times greater than that observed at the soma (Figures 1E–G). While hyperpolarization was conserved at dendritic branches distal to synaptic input, inhibition attenuated steeply at proximal dendritic locations. This is due to the asymmetrical cable properties of neurons: the smaller diameters and “sealed ends” of distal dendrites provide a high resistance that limits voltage attenuation while the soma acts as a sink leading to attenuation in the proximal direction (Jack et al., 1975; Golding et al., 2005). Although our simulations modeled hyperpolarizing inhibition, the shunting action of a distal GABA_A_ synaptic conductance also attenuates steeply towards the soma (Gidon and Segev, 2012). Importantly, simulations using synaptic contacts at locations derived from our cell reconstructions revealed that inhibition could be compartmentalized in a branch-specific fashion within the apical dendrites (Figures 1E,F, 2). Specifically, sparse distal contacts produce the greatest impact on dendritic branches receiving input compared to neighboring un-contacted branches. Indeed, analysis of the spatial extent of inhibition showed that the strongest hyperpolarization is restricted to a very small fraction (<10%) of the apical dendritic tree (Figures 1G, 2). Thus, these simulations predict that a single dendrite-targeting interneuron may have a large local effect on individual branches while producing relatively little change in other dendritic branches or the soma.

**Figure 3:**
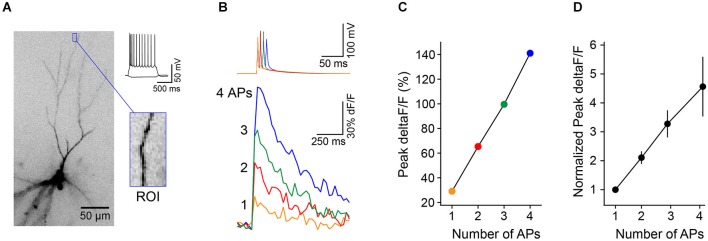
**Brief trains of back-propagating APs cause linear increases in Ca^2+^ in L3 PC distal apical dendrites. (A)** L3 PC (identified by morphology and intrinsic electrical properties) filled with the calcium-sensitive dye OGB1-AM. Blue box, distal region of interest (ROI) used for measurements of Ca^2+^ transients. **(B)** Trains of 1–4 APs (50 Hz) evoked via somatic current injection (top traces) elicit Ca^2+^ transients (bottom traces, deltaF/F) in the ROI from cell in **(A)**. **(C)** The relationship between peak dF/F and number of APs is linear in this range. **(D)** Summary of results (*n* = 8 cells, error bar = ±SEM) shows linear relationship between Ca^2+^ and number of APs.

**Figure 4:**
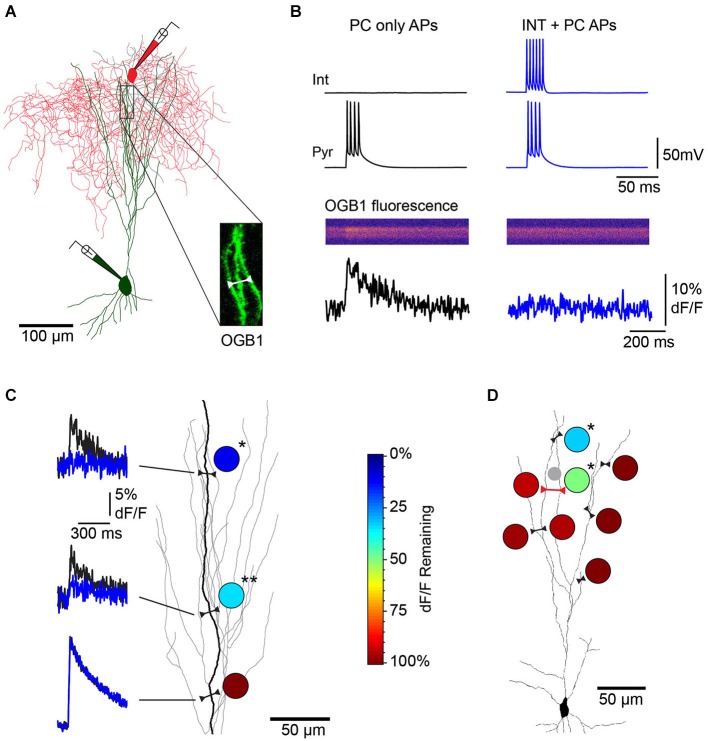
**Single L1 interneurons elicit branch specific inhibition of bAP-evoked Ca^2+^ transients in L3 PC apical dendrites. (A)** Reconstruction of a synaptically connected cell pair from a 2-photon image stack of an Oregon Green-BAPTA1 (OGB1)-filled L3 PC (green) and L1 interneuron (red) filled with Alexa 594 dye. Inset, location of distal apical dendrite line scan. **(B)** Single L1 INT suppresses bAP-evoked Ca^2+^ influx in L3 PC apical dendrites (responses of cell pair and dendrite region from **(A)**). Left, dendritic Ca^2+^ transient (bottom) elicited by PC somatic APs (top, black traces) under control conditions. Right, a train of APs in the connected L1 INT abolishes the bAP-evoked Ca^2+^ transient in the PC dendrite (blue traces). **(C)** Multiple line scans over different regions of the same PC dendrite branch show location-dependent suppression of bAP-evoked Ca^2+^ responses. Left, dF/F elicited by PC only APs (black) overlaid with dF/F elicited by INT+PC APs (blue). Circles, heat map of dendritic inhibition plotted as percent of control dF/F remaining during INT co-activation at each of the three imaging sites. (*p = 0.0103, **p = 0.0066; *n* = 4 trials/condition). Color scale on right applies to all other panels. **(D)** Multiple line scans over different dendritic branches of the same PC show branch-specific inhibition. Single line scan including two neighboring branches (red line) confirmed branch-specific modulation of Ca^2+^ transients during the same trials. (*p = 0.0414 (proximal site), *p* = 0.0206 (distal site); *n* = 3 trials/condition). The small gray circle represents the location of the L1 INT soma.

To test whether individual dendrite-targeting interneurons elicit branch-specific inhibition, we measured PC dendrite excitability by recording Ca^2+^ transients produced by back-propagating action potentials (bAPs). We evoked brief bursts of somatic APs (3–5 APs at 100 Hz) in PCs filled with the Ca^2+^ indicator Oregon Green-BAPTA1 and used 2-photon imaging to map bAP-evoked Ca^2+^ transients in apical dendrites (Figures 4A,B). Piriform PC dendrites do not elicit regenerative Ca^2+^ spikes and the relationship between dendritic Ca^2+^ influx and the number of bAPs is linear within this range (Bathellier et al., 2009; Johenning et al., 2009; Figure 3). Under these conditions, somatic AP bursts elicited robust Ca^2+^ transients even in the most distal dendrites of PCs (Figure 4B, left). On interleaved trials, PC APs were delivered together with a train of APs to a synaptically connected L1 INT (average unitary synaptic conductance 0.19 ± 0.02 nS, *n* = 23). We found that activation of a single interneuron could largely abolish bAP-evoked dendritic Ca^2+^ transients (Figure 4B, right). While experiments using trains of APs in both the L1 INT and the PC are summarized here, we were able to confirm that a single L1 INT AP was capable of modulating dendritic bAP-evoked Ca^2+^ transients, and that Ca^2+^ transients generated by single somatic APs in the PC could be suppressed by L1 INT firing (not shown).

**Figure 5:**
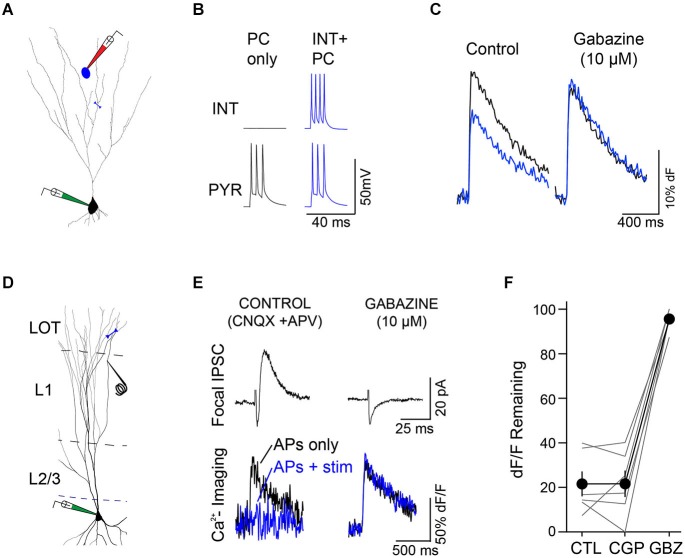
**Modulation of dendritic excitability is mediated by GABA_A_ receptors. (A)** Synaptically-connected L1 interneuron (blue)-L3 PC (black) pair. Blue line indicates region of line scan for measurement of Ca^2+^ transients **(B)** PC APs alone (black traces) were interleaved with trials in which APs were evoked in both the interneuron and PC (blue traces). **(C)** Under control conditions (left) co-activation of the L1 interneuron (blue trace) caused a marked reduction in the PC bAP-evoked Ca^2+^ transient (black trace). Right: application of gabazine (10 uM) completely blocked the suppression of the bAP-evoked Ca^2+^ transient by the L1 interneuron. **(D)** Minimal stimulation experiments confirmed the role of GABA_A_ receptors in the modulation of bAP-evoked Ca^2+^ transients. Experimental configuration, whole-cell recordings were made from L3 PCs filled with OGB1-AM. A focal stimulating electrode was placed close to a branch of dendrite where line scans (blue line) were performed. Experiments were performed in the presence of glutamate receptor antagonists (CNQX, 10 μM and D-APV, 50 μM) to isolate inhibitory synaptic transmission. **(E)** Representative experiment. Left, top: focal stimulation elicited a small inhibitory postsynaptic current (IPSC (black trace, *V*_m_ = −45 mV) in the PC. Bottom: bAP-evoked Ca^2+^ transients in the PC (black trace) were strongly reduced during a train (6 pulses, 100 Hz) of L1 stimuli (blue traces). Right, application of gabazine (10 μM) eliminated the L1-evoked IPSC (top) and abolished the effect of L1 stimulation on the PC bAP-evoked Ca^2+^ transients. **(F)** Summary (*n* = 6 cells, lines represent individual cells, circles indicate mean ± SEM) demonstrating that suppression of bAP-evoked Ca^2+^ transients by focal L1 stimulation is unaffected by the GABA_B_ receptor antagonist CGP 55845 (10 μM, CGP) but completely blocked by subsequent application of gabazine (10 μM, GBZ).

Imaging multiple regions of the same dendritic branch revealed that the suppression of dendritic Ca^2+^ transients was highly localized along the branch. Specifically, we observed that while distal dendritic sites were strongly inhibited, more proximal locations on the same dendritic branch were much less affected (Figure 4C). We further found that the impact of single L1 INTs was quite heterogeneous across different branches of the same PC dendrite; while Ca^2+^ transients in individual branches were markedly suppressed, responses in other branches equidistant from the soma were completely unaffected (Figure 4D). The suppression of bAP-evoked Ca^2+^ transients was abolished by gabazine, indicating that the effects of L1 interneurons were due to GABA_A_ receptor-mediated inhibition (Figures 5A–C, *n* = 2). To further examine this, we directly tested the relative contribution of GABA_A_ and GABA_B_ receptors in the suppression of bAP-evoked Ca^2+^ by dendritic inhibition produced with focal stimulation, and found that only blockade of GABA_A_ receptors affected the magnitude of suppression (Figures 5D–F).

**Figure 6:**
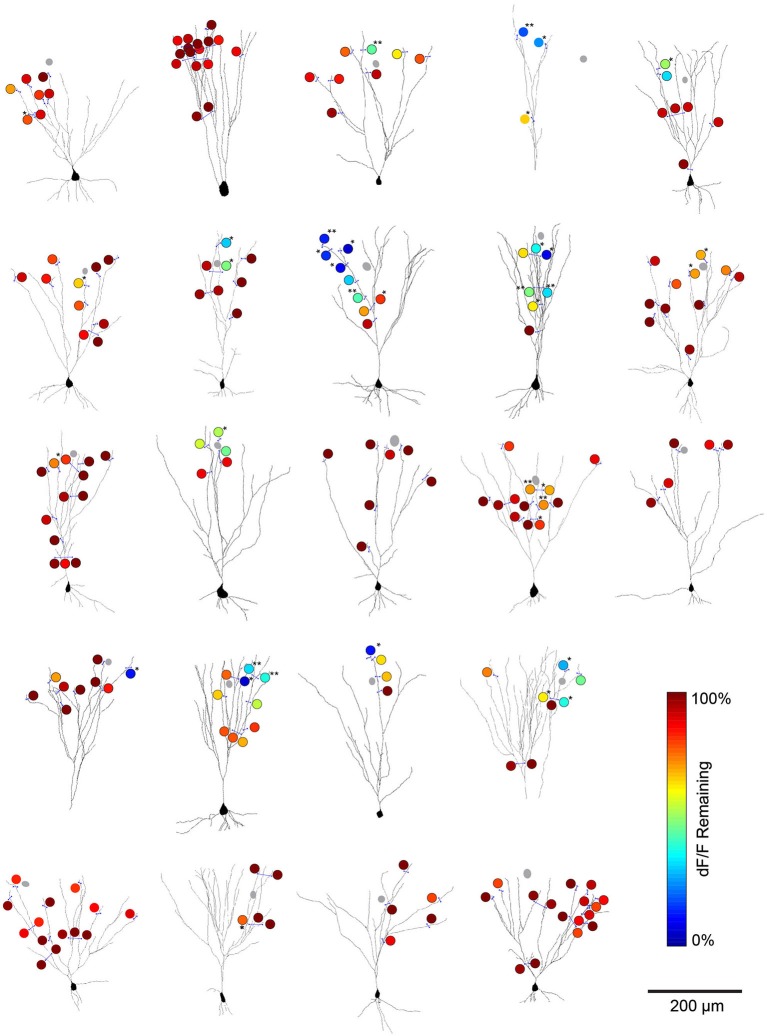
**L1 interneuron-mediated modulation of bAP-evoked Ca^2+^ transients for all imaged cell pairs**. Summary plots of imaging results for the cell pairs analyzed in Figure 2. Cells were reconstructed from 2-photon stacks of OGB1-AM raw fluorescence in L3 PCs. Blue lines represent the location of line scans used for deltaF/F calculations. Gray circles represent the soma of the simultaneously recorded L1 interneuron. The color scale (circles) indicates the percent of the peak deltaF/F remaining when the L1 interneuron was co-activated with the L3 PC (compared to the Ca^2+^ response when bAPs were evoked in L3 PCs alone). Significance calculated by student’s *t*-test comparing peak deltaF/F values for trials of PYR APs alone vs. trials of INT+PYR APs (3–5 trials per condition per imaging site), *=*p* < 0.05; **=*p* < 0.01.

**Figure 7:**
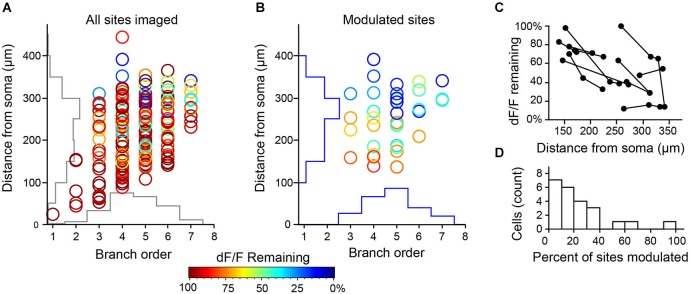
**Summary of modulation at all imaging sites for L1 interneuron-PC pairs. (A)** (*N* = 201 sites, 23 cell pairs from 23 slices and 22 mice). Axes show corresponding histograms of imaging locations. **(B)** Distribution of sites with significant modulation (36 sites in 17 cell pairs, *p* < 0.05, 3–5 trials/condition) show strongest effect on most distal dendrites. **(C)** When multiple sites were imaged along the same dendritic branch (connected points), the effect of inhibition increased with distance from the soma. **(D)** Inhibition by single L1 INTs operates over a small fraction of the total dendritic arbor. Histogram represents the percentage of imaged sites in a single L3 PC with significant modulation by an individual L1 INT.

We pooled data from all cell pairs (Figure 6) to quantify the spatial extent of dendritic inhibition elicited by individual L1 interneurons. We found that Ca^2+^responses at most imaged dendritic sites of PCs (82%, *N* = 201 sites, 23 cells) were unaffected by activation of the connected interneurons (Figure 7A). At sites where interneuron input significantly modulated the Ca^2+^ transient, the peak deltaF/F remaining in the presence of inhibition ranged from 7–83% of the control response (mean = 45%, *N* = 36 sites; Figure 7B). Inhibition was only observed at dendritic sites >100 μm (mean = 251 ± 11μm) from the soma and the strongest suppression of bAP-evoked Ca^2+^ transients (>50% reduction in dF/F) was observed at the most distal dendritic sites (>250 μm from soma) regardless of branch order (Figure 7B). Furthermore, inhibition of Ca^2+^ responses was typically graded with distance from the soma when multiple sites were imaged along the same dendritic branch (Figure 7C). Overall, while we observed inhibition at dendritic sites in the majority of PCs tested (17/23), modulation was sparse and occurred at only a small fraction of imaged sites (mean = 22 ± 5%) in each cell (Figure 7D). Given that we oversampled dendritic sites along branches in which we observed modulation (i.e., Figure 6), the fraction of the total apical dendritic tree inhibited by a single interneuron is likely much sparser.

**Figure 8:**
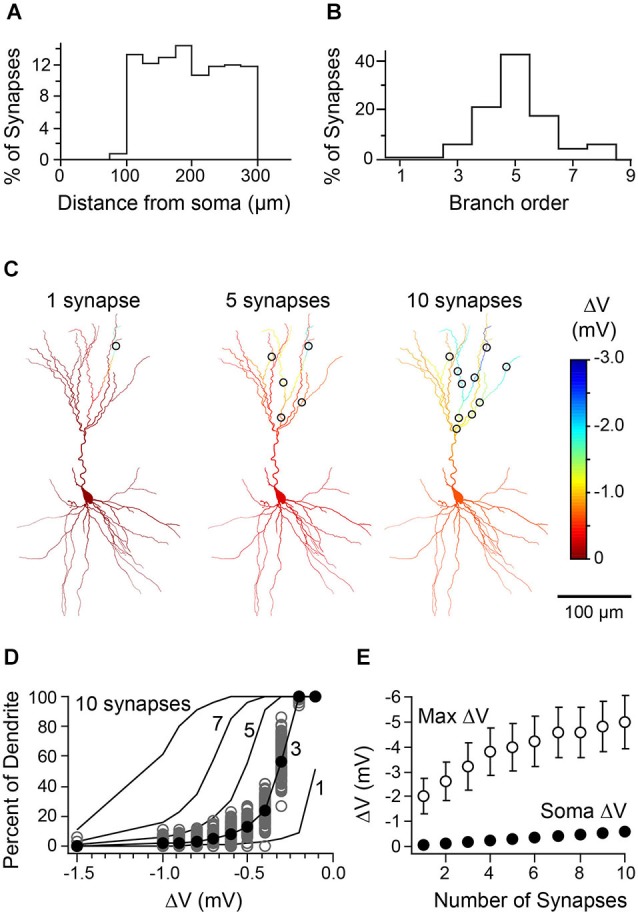
**Relationship between number of synaptic contacts of L1 interneuron-L3 PC pairs and distribution of inhibition. (A)** Histogram representing the distance from soma for locations of synapses drawn in the model (*n* = 100 synapses/cell over 5 cells; compare to histogram in Figure 1C). **(B)** Histogram of the branch order for synapses randomly drawn from the uniform distribution represented in Figure 1D. **(C)** Simulated spatial profile of hyperpolarization from the activation of 1, 5, or 10 randomly placed synapses (same cell as in Figure 1). **(D)** Cumulative distribution curves representing mean dendritic hyperpolarization generated by 1, 3, 5, 7, or 10 synapses placed in the same cell. Gray circles, 100 iterations of 3 synapses distributed over the apical dendrites. Black circles, the mean cumulative distribution of dendritic hyperpolarization (mean of values depicted by gray circles). Black lines, mean curves generated by 100 iterations of the model. **(E)** Summary data from simulations of five cells showing the mean maximal dendritic voltage change (open circles) and somatic voltage change (closed circles) as a function of the number of synapses activated (error bars are < SEM).

What accounts for the compartmentalization of inhibition we observe? We believe it reflects a combination of the sparseness and distribution of inhibitory synaptic contacts as well as the cable properties of PCs. To test this idea, we simulated the simultaneous activation of 1–10 synapses spread throughout the apical dendrite to determine how synapse number would affect the spatial extent of inhibition. Synapses were placed semi-randomly such that their location matched the statistics of putative synapses from our biocytin reconstructions (Figures 8A,B). Indeed, as expected, progressively adding distal synapses led to a more widespread hyperpolarization throughout the dendritic tree (Figures 8C,D) and although the somatic voltage change remained quite weak (<1 mV), maximal dendritic inhibition was substantially increased (Figure 8E). Thus, sparse synaptic contacts and the membrane properties of PCs are likely to be the dominant factors generating branch-specific inhibition.

## Discussion

Here we combine 2-photon imaging of PC dendritic excitability with paired recordings to investigate the spatial domain of inhibition of individual dendrite-targeting interneurons. We show that L1 interneurons make sparse contacts onto the distal apical dendrites of L3 PCs in piriform cortex. The sparse contacts made by individual interneurons dampen the propagation of bAPs, and thus dendritic excitability, in a branch specific fashion. Our experiments focused on acute slices made from animals younger than 4 weeks old; because bAPs have been proposed to play an important role in both the development and mature function of cortical microcircuits (Häusser and Mel, 2003; Johenning et al., 2009; Smith et al., 2013), it will be critical to determine whether these findings are also observed in adult animals and whether this mode of inhibition is integral to the function of the intact circuit.

Although previous studies have used optogenetic activation of GABAergic boutons or GABA uncaging along with calcium imaging to probe the spatial domain of dendritic inhibition (Kanemoto et al., 2011; Chiu et al., 2013), very few studies have examined the impact of a single dendrite-targeting interneuron on the dendritic excitability of its postsynaptic partner. Direct dendritic patch clamp recording revealed that activation of a single interneuron could block dendritic Ca^2+^ spikes in neocortical layer 5 PCs (Larkum et al., 1999), however, the locations of the interneuron contacts and whether the inhibitory effect was global or focal were not established. Our findings are consistent with recent work suggesting that GABA inputs act focally (Kanemoto et al., 2011; Chiu et al., 2013). However, in contrast to the proposed dendritic spine-specific action of somatostatin-expressing (SOM) interneurons (Chiu et al., 2013) our results using lower magnification line scans suggest that L1 interneurons in piriform cortex suppress the excitability of the dendritic shaft. This may reflect the selective targeting of SOM cell synapses to dendritic spines (Chiu et al., 2013), while L1 interneurons could synapse onto the dendritic shaft.

We determined that L1 interneurons make sparse connections on PCs by using light microscopy to estimate synaptic contacts from biocytin-filled cells in brain slices. Although this approach based on the close apposition of axons and dendrites has been routinely used to identify putative synaptic contacts (Miles et al., 1996; Markram et al., 1997; Feldmeyer et al., 2006), it is possible this approach overestimates the true number of synaptic contacts. Additionally, it is also possible that slicing artifact has reduced the complexity of axonal branching and therefore decreased the apparent number of synaptic connections in both the anatomic and functional data. We sought to compensate for these two possibilities by only including cell pairs for analysis with high-quality cell fills, grossly intact structure and significant (>50%) overlap between interneuron axon and PC dendrite fields based on cell fills. Nonetheless, even without electron microscopic confirmation of each putative synaptic contact, our data strongly suggest that single L1 interneurons make relatively few synapses onto L3 PCs. In future studies, it will be important to directly compare the sites of synaptic contact with the modulation of dendritic excitability to establish the “space constant” over which a single inhibitory dendritic synapse acts.

Both the conductance shunt and hyperpolarization provided by GABA_A_ receptors may contribute to the inhibition of dendritic Ca^2+^ transients (Tsubokawa and Ross, 1996). We found that dendritic hyperpolarization caused by distal inhibitory synapses is quite localized, consistent with the spatial suppression of bAP-associated Ca^2+^ transients. The amplitude of bAPs in the distal dendrites of PCs in piriform cortex can be attenuated up to 50% due to the passive properties of these dendrites (Bathellier et al., 2009), thus relatively small changes in voltage may be sufficient to effect large modulation in the amplitude of bAP-associated Ca^2+^ transients. Alternately, recent modeling studies have shown that the shunting effects of inhibition can be observed both locally and globally (Gidon and Segev, 2012). It is possible that in a system with sparsely distributed inhibitory synaptic contacts the local effects of shunting predominate. In this study we were not able to directly test the relative contribution of conductance shunt vs. hyperpolarization to the suppression of bAP-associated Ca^2+^ transients.

Studies in sensory cortex have debated whether the spatial patterning of excitatory synaptic input and active dendritic compartments contribute to information coding (Häusser and Mel, 2003; Jia et al., 2010; Smith et al., 2013). Our results indicate that individual interneurons can selectively inhibit particular PC dendritic branches in piriform cortex; this is a consequence of both the sparseness and location of L1 INT synapses as well as the cable properties of L3 PCs. Our findings provide additional evidence that GABAergic signaling in the dendrites of PCs exhibits a remarkable level of spatial precision (Higley, 2014). While future studies will be necessary to determine the implications of localized GABAergic inhibition on cortical circuit function, these findings suggest that individual inhibitory interneurons may play a significant role in shaping the spatial integration of sensory information in the dendrites of PCs.

## Conflict of interest statement

The authors declare that the research was conducted in the absence of any commercial or financial relationships that could be construed as a potential conflict of interest.

## References

[B1] Bagnall M. W. Hull C. Bushong E. A. Ellisman M. H. Scanziani M. (2011). Multiple clusters of release sites formed by individual thalamic afferents onto cortical interneurons ensure reliable transmission. Neuron 71, 180–194. 10.1016/j.neuron.2011.05.032 21745647 PMC3271052

[B2] Bathellier B. Margrie T. W. Larkum M. E. (2009). Properties of piriform cortex pyramidal cell dendrites: implications for olfactory circuit design. J. Neurosci. 29, 12641–12652. 10.1523/JNEUROSCI.1124-09.2009 19812339 PMC6665100

[B3] Chiu C. Q. Lur G. Morse T. M. Carnevale N. T. Ellis-Davies G. C. R. Higley M. J. (2013). Compartmentalization of GABAergic inhibition by dendritic spines. Science 340, 759–762. 10.1126/science.1234274 23661763 PMC3752161

[B4] Cobb S. R. Buhl E. H. Halasy K. Paulsen O. Somogyi P. (1995). Synchronization of neuronal activity in hippocampus by individual GABAergic interneurons. Nature 378, 75–78. 10.1038/378075a0 7477292

[B5] Feldmeyer D. Lübke J. Sakmann B. (2006). Efficacy and connectivity of intracolumnar pairs of layer 2/3 pyramidal cells in the barrel cortex of juvenile rats. J. Physiol. 575, 583–602. 10.1113/jphysiol.2006.105106 16793907 PMC1819447

[B6] Gidon A. Segev I. (2012). Principles governing the operation of synaptic inhibition in dendrites. Neuron 75, 330–341. 10.1016/j.neuron.2012.05.015 22841317

[B7] Golding N. L. Mickus T. J. Katz Y. Kath W. L. Spruston N. (2005). Factors mediating powerful voltage attenuation along CA1 pyramidal neuron dendrites. J. Physiol. 568, 69–82. 10.1113/jphysiol.2005.086793 16002454 PMC1474764

[B8] Häusser M. Mel B. (2003). Dendrites: bug or feature?. Curr. Opin. Neurobiol. 13, 372–383. 10.1016/s0959-4388(03)00075-8 12850223

[B9] Higley M. J. (2014). Localized GABAergic inhibition of dendritic Ca^2+^ signalling. Nat. Rev. Neurosci. 15, 567–572. 10.1038/nrn3803 25116141 PMC4383162

[B10] Jack J. J. B. Noble D. Tsien R. W. (1975). Electric Current Flow in Excitable Cells. Oxford: Clarendon Press.

[B11] Jia H. Rochefort N. L. Chen X. Konnerth A. (2010). Dendritic organization of sensory input to cortical neurons in vivo. Nature 464, 1307–1312. 10.1038/nature08947 20428163

[B12] Johenning F. W. Beed P. S. Trimbuch T. Bendels M. H. K. Winterer J. Schmitz D. (2009). Dendritic compartment and neuronal output mode determine pathway-specific long-term potentiation in the piriform cortex. J. Neurosci. 29, 13649–13661. 10.1523/JNEUROSCI.2672-09.2009 19864577 PMC6664992

[B13] Kanemoto Y. Matsuzaki M. Morita S. Hayama T. Noguchi J. Senda N.. (2011). Spatial distributions of GABA receptors and local inhibition of Ca2+ transients studied with GABA uncaging in the dendrites of CA1 pyramidal neurons. PLoS One 6:e22652. 10.1371/journal.pone.0022652 21799926 PMC3143187

[B14] Larkum M. E. Zhu J. J. Sakmann B. (1999). A new cellular mechanism for coupling inputs arriving at different cortical layers. Nature 398, 338–341. 10.1038/18686 10192334

[B15] Lovett-Barron M. Turi G. F. Kaifosh P. Lee P. H. Bolze F. Sun X.-H.. (2012). Regulation of neuronal input transformations by tunable dendritic inhibition. Nat. Neurosci. 15, 423–430, S1–S3. 10.1038/nn.3024 22246433

[B16] Markram H. Lübke J. Frotscher M. Roth A. Sakmann B. (1997). Physiology and anatomy of synaptic connections between thick tufted pyramidal neurones in the developing rat neocortex. J. Physiol. 500(Pt. 2), 409–440. 9147328 10.1113/jphysiol.1997.sp022031 PMC1159394

[B17] Markram H. Toledo-Rodriguez M. Wang Y. Gupta A. Silberberg G. Wu C. (2004). Interneurons of the neocortical inhibitory system. Nat. Rev. Neurosci. 5, 793–807. 10.1038/nrn1519 15378039

[B18] Miles R. Tóth K. Gulyás A. Hájos N. Freund T. (1996). Differences between somatic and dendritic inhibition in the hippocampus. Neuron 16, 815–823. 10.1016/S0896-6273(00)80101-4 8607999

[B19] Müller C. Beck H. Coulter D. Remy S. (2012). Inhibitory control of linear and supralinear dendritic excitation in CA1 pyramidal neurons. Neuron 75, 851–864. 10.1016/j.neuron.2012.06.025 22958825

[B20] Smith S. L. Smith I. T. Branco T. Häusser M. (2013). Dendritic spikes enhance stimulus selectivity in cortical neurons in vivo. Nature 503, 115–120. 10.1038/nature12600 24162850 PMC6319606

[B21] Stokes C. C. A. Isaacson J. S. (2010). From dendrite to soma: dynamic routing of inhibition by complementary interneuron microcircuits in olfactory cortex. Neuron 67, 452–465. 10.1016/j.neuron.2010.06.029 20696382 PMC2922014

[B22] Suzuki N. Bekkers J. M. (2012). Microcircuits mediating feedforward and feedback synaptic inhibition in the piriform cortex. J. Neurosci. 32, 919–931. 10.1523/JNEUROSCI.4112-11.2012 22262890 PMC6621151

[B23] Tsubokawa H. Ross W. N. (1996). IPSPs modulate spike backpropagation and associated [Ca2+]i changes in the dendrites of hippocampal CA1 pyramidal neurons. J. Neurophysiol. 76, 2896–2906. 8930242 10.1152/jn.1996.76.5.2896

